# Supplementary material for: Integrated analysis of the aqueous humor microbiome and lens capsule transcriptome in high myopia cataract: a pilot study
Source: Front Med (Lausanne). 2026 Jun 16;13:1845205. doi: 10.3389/fmed.2026.1845205 (PMC13314463; doi:10.3389/fmed.2026.1845205)
Supplement: Supplementary file 3 [file Data_Sheet_1.zip › 3.Beta_Diversity/NMDS/C372089/NMDS_euclidean_3D.pdf]

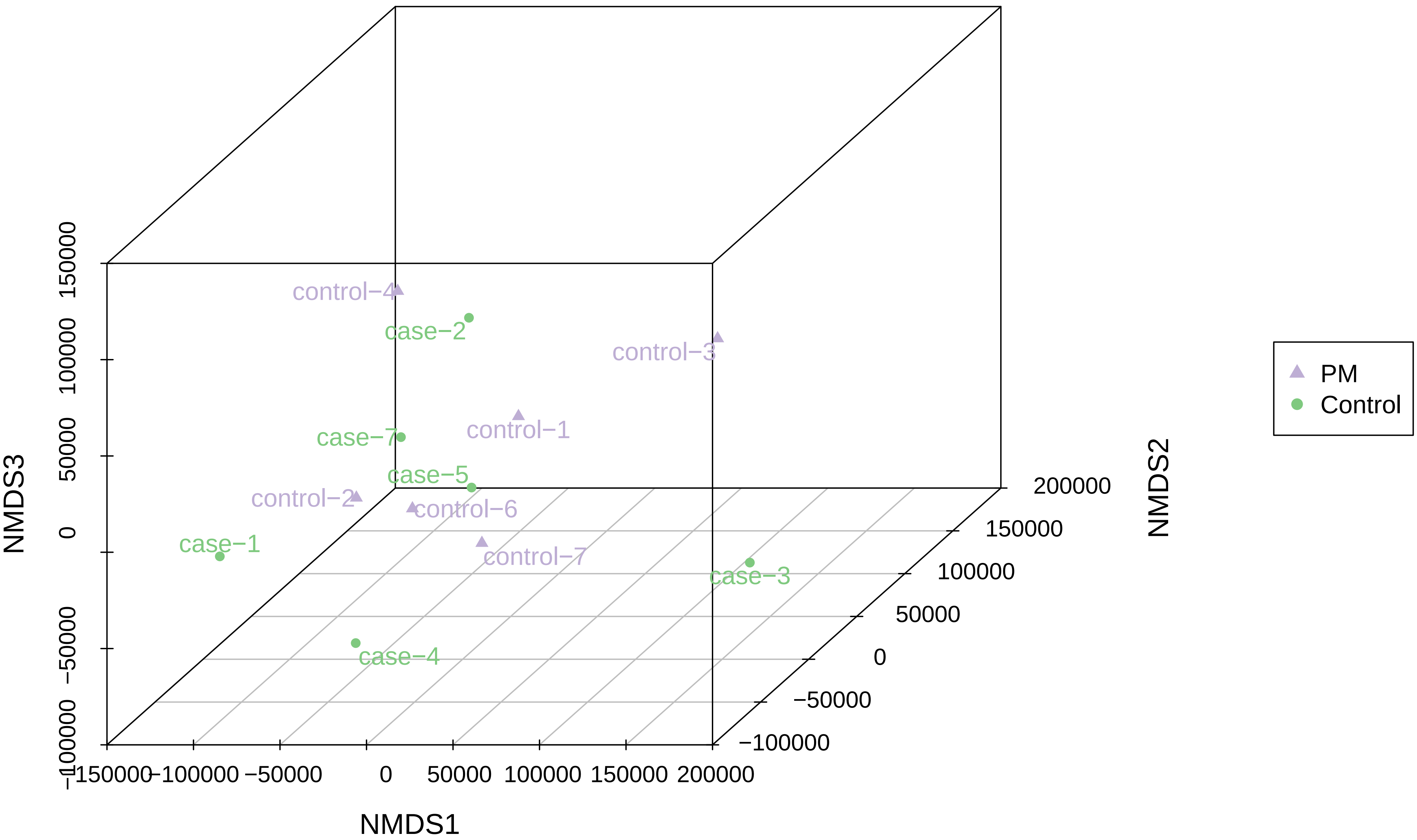

control−4
case−2
control−3
control−1
case−7
case−5
control−2
control−6
case−1
control−7
case−3
case−4
PM
Control
NMDS3
150000
100000
50000
0
−50000
−100000
NMDS1
−150000
−100000
−50000
0
50000
100000
150000
200000
NMDS2
200000
150000
100000
50000
0
−50000
−100000
